# Randomized controlled trial of single incision versus conventional multiport laparoscopic cholecystectomy with long-term follow-up

**DOI:** 10.1007/s00423-020-01911-1

**Published:** 2020-06-29

**Authors:** Denis Klein, Atakan Görkem Barutcu, Dino Kröll, Maik Kilian, Johann Pratschke, Roland Raakow, Jonas Raakow

**Affiliations:** 1 Department of Surgery, Charité Campus Mitte, Campus Virchow Klinikum, Charité – Universitätsmedizin Berlin, corporate member of Freie Universität Berlin, Humboldt-Universität zu Berlin, and Berlin Institute of Health, Charitéplatz 1, 10117 Berlin, Germany; 2 Department of General and Visceral Surgery, Evangelische Elisabeth Klinik, Lützowstraße 26, 10785 Berlin, Germany; 3 Department of General, Visceral and Vascular Surgery, Vivantes Klinikum Am Urban, Dieffenbachstrasse 1, 10967 Berlin, Germany

**Keywords:** Laparoscopic surgery, Single-incision, Single-port, Cholecystectomy, Multiport

## Abstract

**Background:**

Within the last years, single-incision laparoscopic cholecystectomy (SLC) emerged as an alternative to multiport laparoscopic cholecystectomy (MLC). SLC has advantages in cosmetic results, and postoperative pain seems lower. Overall complications are comparable between SLC and MLC. However, long-term results of randomized trials are lacking, notably to answer questions about incisional hernia rates, long-term cosmetic impact and chronic pain.

**Methods:**

A randomized trial of SLC versus MLC with a total of 193 patients between December 2009 and June 2011 was performed. The primary endpoint was postoperative pain on the first day after surgery. Secondary endpoints were conversion rate, operative time, intraoperative and postoperative morbidity, technical feasibility and hospital stay. A long-term follow-up after surgery was added.

**Results:**

Ninety-eight patients (50.8%) underwent SLC, and 95 patients (49.2%) had MLC. Pain on the first postoperative day showed no difference between the operative procedures (SLC vs. MLC, 3.4 ± 1.8 vs. 3.7 ± 1.9, respectively; *p* = 0.317). No significant differences were observed in operating time or the overall rate of postoperative complications (4.1% vs. 3.2%; *p* = 0.731). SLC exhibited better cosmetic results in the short term. In the long term, after a mean of 70.4 months, there were no differences in incisional hernia rate, cosmetic results or pain at the incision between the two groups.

**Conclusions:**

Taking into account a follow-up rate of 68%, the early postoperative advantages of SLC in relation to cosmetic appearance and pain did not persist in the long term. In the present trial, there was no difference in incisional hernia rates between SLC and MLC, but the sample size is too small for a final conclusion regarding hernia rates.

**Trial registration:**

German Registry of Clinical Trials DRKS00012447

## Introduction

Since the first laparoscopic cholecystectomy was performed by Mühe et al. in 1985, significant progress in the practice of minimal invasive gallbladder surgery revolutionized the surgical treatment of benign gallbladder diseases [1]. Conventional multiport laparoscopic cholecystectomy (MLC) is the gold standard, but new techniques were introduced to further minimize the impact of surgery. One-wound laparoscopy, later described as single-incision or single-port laparoscopic surgery (SLS), was first introduced by Navarra et al. in 1997 [2]. Within the last few years, this surgical procedure received increasing attention, and several authors documented the safety and feasibility of SLS in a wide variety of surgical indications [3–6]. SLS has a positive cosmetic effect because of a single transumbilical incision, and it produces less pain and faster recovery due to the reduction of incisional trauma [7–10]. Single-incision laparoscopic cholecystectomy (SLC) is becoming more established as a potential new standard surgery for gallbladder diseases, and numerous reports were published for elective cholecystectomy. Single-incision surgery produces similar results as conventional multiport laparoscopic cholecystectomy, particularly conversion and complication rates. However, long-term results, especially umbilical incisional hernia rates, are lacking and subject to controversy [11–14].

Therefore, we performed a randomized trial to compare single-port with multiport laparoscopic cholecystectomy within the early days of single-port laparoscopy and added a long-term follow-up of the patients with particular focus on the development of incisional hernias and long-term cosmetic results.

## Methods

The study was designed as a prospective randomized clinical trial at a single institution between December 2009 and June 2011. Approval was obtained from the local ethics committee and the institutional review board. The study is registered at the German Registry of Clinical Trials (registration number DRKS00012447). All subjects provided written informed consent prior to inclusion in the study.

The following inclusion criteria were used: age over 18 years and indication for laparoscopic cholecystectomy (symptomatic cholecystolithiasis, cholecystitis or gallbladder polyps). Exclusion criteria included contraindications to laparoscopy, American Society of Anesthesiologists (ASA) class IV or V, pregnancy or lactation.

### Randomization and outcome measurement

All patients were randomized at a 1:1 ratio to SLC or MLC procedure via the drawing of sealed opaque envelopes containing computer-generated random numbers before the start of surgery. A third party prepared the envelopes, and block sizes of two and four were used randomly and unknown to the operating surgeon.

The primary outcome was the measurement of pain on the first postoperative day using a visual analogue scale (VAS). Pain scores were recorded on the morning of the first postoperative day and the day of discharge.

Secondary endpoints were conversion rate, operative time, intraoperative and postoperative morbidity, technical feasibility and hospital stay. The operative time was defined as the time from the skin incision to the complete application of all wound dressings. Intraoperative complications were defined as any adverse event during the surgery that required additional intervention or treatment. Postoperative morbidity was defined as any adverse event that required additional medical or surgical intervention during the short-term follow-up and was classified according to Dindo et al. [15]. The length of hospital stay was the time from admission to discharge, counting the day of admission and operation as day 0. The surgeon who performed the operation documented the technical feasibility immediately after skin closure on a 10-point scale where 1 represented very good feasibility and 10 represented the worst surgical feasibility.

After an initial analysis, we added a long-term follow-up of all patients. Therefore, we added the rate of incisional hernia and cosmetic result of the long-term follow-up to the secondary endpoints.

### Surgical procedures

The surgical techniques for MLC and SLC were standardized. Three experienced laparoscopic surgeons, each of whom performed over 100 ML and more than 50 SIL cholecystectomies, performed or supervized all surgeries. Prophylactic antibiotic therapy in both groups consisted of single-shot cefotaxime (2 g) and metronidazole (500 mg), which were administered intravenously shortly before the skin incision. Patients for MLC and SLC were placed in a supine position with both surgeons standing on the left side of the patient.

Access to the abdominal cavity for SLC procedures was achieved via mini-laparotomy through a single transumbilical 15- to 20-mm skin and fascial incision. A commercial port system (TriPort™ or TriPort+™; Olympus, Japan) was inserted with the aid of the supplied introducer. A 30° 5- or 10-mm laparoscope and two standard straight 5-mm working instruments were inserted through the port system.

The MLC was a three-port approach, and the pneumoperitoneum was established using a Veress needle. A 10-mm trocar was placed subumbilically for the 30° laparoscope, and two working trocars (5 and 10 mm) were inserted under sight epigastrically and in the middle right lateral region.

Calot’s triangle was dissected in all procedures, and the cystic duct and artery were ligated using a 5-mm endoscopic clip applier (Ligamax5 M/L, Ethicon, USA) and divided with scissors. The gallbladder was carefully dissected from the fossa. The gallbladder was removed directly through the port system in SLC procedures, which also acts as a wound protector. Gallbladder extraction for MLC was done via the umbilical incision with a retrieval bag to avoid wound contamination. The umbilical and 10-mm incisions were closed using non-absorbable 0 sutures for the fascial incision and absorbable 4-0 monofilament sutures for skin closure. We did not apply local anaesthetics around the trocar incisions as pain medication.

### Postoperative care and follow-up

All patients received the same standard analgesia prescription during the postoperative care. This care consisted of 1000 mg metamizole intravenously three times daily for the first postoperative day and was reduced to 500 mg metamizole orally three times daily until discharge. A rescue medication of 50 mg tramadol orally was titrated up to three times daily on demand until the VAS pain score was lower than 3. Patients were discharged around the second day after surgery as soon as oral feeding was tolerated, and there were no signs of fever or uncontrolled pain.

The follow-up was started in August 2016 via letter correspondence with the patients. The questionnaire included information on clinical symptoms that suggest incisional hernia, information on any other abdominal surgery after the cholecystectomy and questions about the patient’s cosmetic opinion of the scar and pain or discomfort at the scar. If no response was received by the end of 2016, patients were contacted by telephone up to three times with at least 1 week in between contact attempts. If no response was achieved, patients were counted as lost to follow-up due to no contact. All patients were asked the same questions to detect an incisional hernia according to the findings of Baucom et al. [16]. In cases of further abdominal surgery after the cholecystectomy, more information about the diagnosis and the extent of the surgery was obtained to exclude any manipulation at the access sites of the cholecystectomy. Contacted patients, especially patients with inconclusive results after the letter and/or telephone contact, were invited for a clinical examination. Clinical examination consisted of palpatory evaluation at rest and under strain and dynamic ultrasound imaging of the laparoscopic access sites. Patients who were converted to an open surgery during SLC or MLC were excluded from the follow-up. The time of letter response, telephone or clinical contact was marked as the follow-up date. The latest date was recorded in cases of multiple contacts. Patients who underwent a further abdominal surgery after the cholecystectomy had their follow-up date recorded as the date of that particular surgery. Patient’s overall opinion of the scar was also measured on a scale ranging from 1 to 10 where patients were asked to rate their scar between “like normal skin” (1) and “very different” (10).

### Statistical analysis

All patient data, including clinical history, demographic factors, laboratory data, indication and details of the surgical procedure, intraoperative findings and postoperative short- and long-term outcomes, were prospectively recorded in a specific database.

The hypothesis was that SLC was associated with less postoperative pain compared with MLC as measured using a VAS score. The sample size calculation was performed before the study was initiated. To identify a difference of 1 point in VAS score with an estimated standard deviation of 2, it was calculated that 84 patients were required per group with a power of 90% and a type 1 error of 5%. With the assumption of at least 10% patient loss to follow-up or dropout in each group, a total sample size of approximately 190 patients was planned for inclusion.

All analyses were based on the intention-to-treat principle, and losses were not replaced. Variables are described as numbers with percentages as appropriate or as means ± standard deviation. Categorical variables were compared using the chi-squared test (*χ*^2^). Numerical continuous variables were compared using the Mann-Whitney *U* test. A *p* value < 0.05 was considered statistically significant. All analyses were performed using SPSS (Statistical Product and Service Solutions) version 23 (IBM, USA).

## Results

**Fig. 1:**
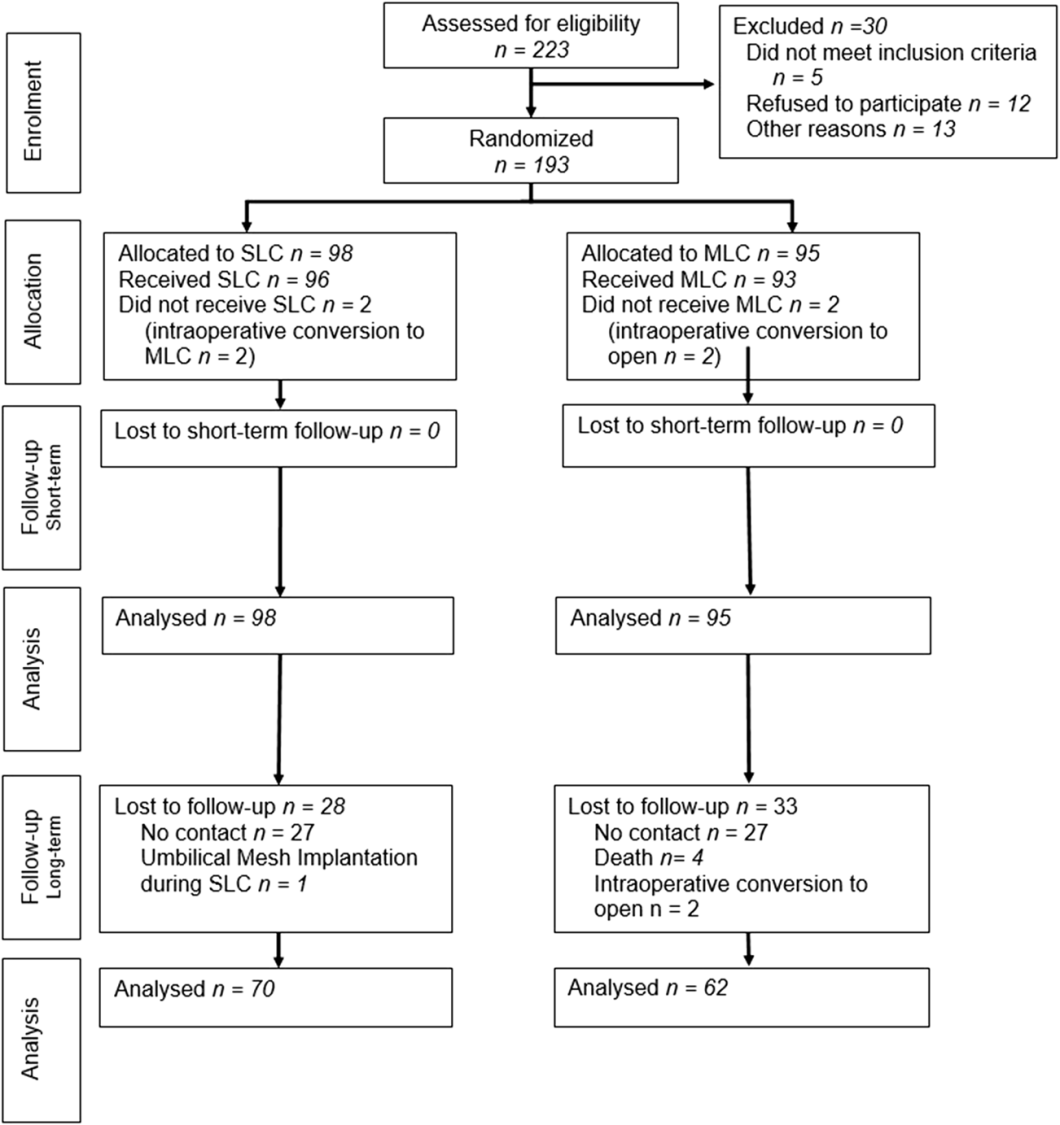
Consort flow diagram

A total of 193 patients (female 66.8%, *n* = 129), aged 19 to 89 years (mean 47.1 years ± 15.4 years), were randomly assigned to SLC (*n* = 98) or MLC (*n* = 95) (Fig. 1). As shown in Table 1, there was no statistical significance between the two groups in demographic factors, such as age, gender, weight, score of the ASA or previous abdominal surgeries.

**Table 1:** Demographic parameters

	SLC (*n* = 98, 50.8%)	MLC (*n* = 95, 49.2%)	*p* value
Gender			0.093
Male	27 (27.6%)	37 (38.9%)	
Female	71 (72.4%)	58 (61.1%)	
Age (years)	45.7 ± 14.9	48.7 ± 15.8	0.213
BMI (kg/m^2^)	28.4 ± 6.0	28.8 ± 6.2	0.835
ASA score			0.083
I	50 (51.0%)	34 (36.2%)	
II	42 (42.9%)	49 (52.1%)	
III	6 (6.1%)	11 (11.7%)	
Previous abdominal surgery	23 (23.5%)	30 (31.6%)	0.207
Diagnosis			0.261
Chronic cholecystitis and others	87 (88.8%)	79 (83.2%)	
Acute cholecystitis	11 (11.2%)	16 (16.8%)	

Values are presented as numbers and percentage or in means ± standard deviation

*BMI* body mass index, *ASA* American Society of Anesthesiologists

Operative times were comparable between groups (Table 2). Intraoperative complications occurred in two patients. Bleeding occurred in one patient undergoing SLC, and a common bile duct injury was found in a patient during MLC, which was treated via suturing and insertion of a T-tube. Two patients in the SLC group needed an additional 5-mm trocar in the epigastrium for better exposure of the hepatoduodenal ligament. No patient in this group was converted to open cholecystectomy. Two patients planned for an MLC were converted to an open procedure due to extensive adhesions.

**Table 2:** Operative details

	SLC (*n* = 98, 50.8%)	MLC (*n* = 95, 49.2%)	*p* value
Operative time (minutes)	55.6 ± 18.3	57.0 ± 17.4	0.430
Peritoneal adhesions	33 (33.7%)	45 (47.4%)	0.058
Gallbladder perforation	17 (17.3%)	24 (25.3%)	0.179
Intraoperative complication	1 (1.0%)	1 (1.1%)	0.368
Conversion			0.975
Multiport	2 (2.0%)	–	
Open	–	2 (2.1%)	
Postoperative complications	4 (4.1%)	3 (3.2%)	0.731
Haematoma/seroma gallbladder fossa	2 (2.0%)		
Haematoma umbilical port	1 (1.0%)	1 (1.1%)	
Epigastric pain/vomiting		1 (1.1%)	
Pneumonia	1 (1.0%)		
Cholestasis		1 (1.1%)	
Clavien-Dindo			0.143
< 3	4 (4.1%)	1 (1.1%)	
≥ 3	–	2 (2.1%)	
Pain at postoperative day 1 (VAS)	3.4 ± 1.8	3.7 ± 1.9	0.317
Pain at discharge (VAS)	1.5 ± 1.3	2.1 ± 1.5	0.021
Hospital stay (days)	3.1 ± 1.2	4.2 ± 2.4	< 0.001
Histological findings			
Chronic inflammation	79 (80.6%)	75 (78.9%)	0.151
Acute inflammation	8 (8.2%)	14 (14.7%)	0.773
Gallstones	75 (76.5%)	74 (77.9%)	0.821

Values are presented as numbers and percentage or in means ± standard deviation

*VAS* visual analogue scale

Surgeons found the single-incision access to be slightly more difficult to perform than the multiport access, but this difference was not statistically significant. The feasibility of the preparation of the gallbladder hilus and fossa was scored similarly in the two groups (Table 3).

**Table 3:** Surgical feasibility

	SLC (*n* = 98, 50.8%)	MLC (*n* = 95, 49.2%)	*p* value
Access	1.4 ± 0.6	1.1 ± 0.3	0.078
Preparation gallbladder hilus	1.9 ± 0.8	1.8 ± 0.7	0.758
Preparation gallbladder fossa	1.7 ± 0.7	1.7 ± 0.8	0.871

Values are presented as numbers and percentage or in means ± standard deviation

Postoperative complications occurred in four patients after SLC and in three patients after MLC (4.1% vs. 3.2%, respectively; *p* = 0.731). A haematoma/seroma in the gallbladder fossa was found in two patients, two patients showed an umbilical haematoma, and one patient developed pneumonia. All of these postoperative complications were grade I or II (Clavien-Dindo). One patient reported prolonged epigastric pain and vomiting, and this patient underwent a gastroscopy without a diagnosis. However, the symptoms improved the day after the intervention. Another patient showed postoperative cholestasis with an endoscopic retrograde cholangiopancreatography that showed swelling of the ductus choledochus, and a papillotomy was performed. These two complications were classified as grade IIIa (Clavien-Dindo).

According to the VAS, pain was similar between the two groups on the morning of the first postoperative day (Table 2), but it was significantly better after SLC on the day of discharge (1.5 ± 1.3 vs. 2.1 ± 1.5; *p* = 0.021). The postoperative hospital stay was significantly shorter after SLC than after MLC (3.1 ± 1.2 days vs. 4.2 ± 2.4 days, respectively; *p* < 0.001).

A total of 68.4% of all patients completed the follow-up after a mean time of 70.4 months. The shortest follow-up was noted after 3 months, and the longest follow-up was 90 months after the operation. Reasons for loss to follow-up are given in Table 4. The overall rate for incisional hernias was 6.8% (9/132), with no difference between SLC and MLC (5.7% vs. 8.1%, respectively; *p* = 0.593). The incisional hernia rates in an intention-to-treat analysis were 5.6% (4/71) after SLC and 7.8% (5/64) following MLC (*p* = 0.735). All hernias in both groups were found at the umbilical incision.

**Table 4:** Follow-up

	SLC (*n* = 98, 50.8%)	MLC (*n* = 95, 49.2%)	*p* value
Follow-up available	70 (71.4%)	62 (65.3%)	0.439
Follow-up time (months)	71.1 ± 16.1	69.6 ± 16.6	0.539
Reasons for loss to follow-up			
No contact	27 (27.6%)	27 (28.4%)	
Death	–	4 (4.2%)	
Umbilical mesh implantation at cholecystectomy	1 (1.0%)	–	
Conversion to open cholecystectomy	–	2 (2.1%)	
Incisional hernia	4 (5.7%)	5 (8.1%)	0.593
Incisional hernia repair	4 (5.7%)	4 (7.3%)	
Open mesh implantation	2	4	
Laparoscopic IPOM	2	–	
Overall cosmetic opinion of the scar	1.5 ± 0.7	1.7 ± 1.8	0.401
Pain at the scar	1.5 ± 0.8	1.2 ± 0.8	0.141

Values are presented as numbers and percentage or in means ± standard deviation

*IPOM* intraperitoneal onlay mesh)

## Discussion

After laparoscopic cholecystectomy had found its way into daily surgical practice, an even further minimization of surgical trauma was achieved using single-incision laparoscopy. The single incision in the navel enables a concealing of the surgical scar by taking advantage of the navel’s natural scar. However, the technique is controversial. Several studies demonstrated the safety and feasibility advantages of single-incision cholecystectomy and highlighted other benefits of this technique [8, 17–20]. However, other studies suggested a higher complication rate and, in particular, an increased incidence of incisional hernias [7, 9, 11]. Most of these studies suffered from the disadvantage of short follow-up observation periods. The present study fills this gap with mean follow-up durations of 71.1 months in the SLC and 69.6 months in the MLC group.

The duration of the surgery is a key parameter for comparing surgical methods. No statistically significant differences were observable in this study. While several earlier studies support this result [8, 20–22], most authors indicate a longer surgery duration [17–19, 23–25] in SLC cases. Several meta-analyses support a longer operative duration [7, 9, 26]. However, all of these meta-analyses reported significant heterogeneity between the observed studies and potential bias effects of a variety of different factors on the time required for surgical completion. Among these factors are the physical condition of the patients, the type of device used in the surgery and the experience of the surgeon. Omar et al. observed a significantly lower duration of the surgery for MLC in a retrospective RCT but also noticed variance in surgery duration during the study period. The first ten SLCs in his study had a duration of 103 min whereas the last ten cases showed a similar duration to the MLC group of 47 min [19]. Other studies also found a decreasing duration of surgeries with an increasing experience of surgeons [23, 27]. This result suggests that the duration of the surgery highly depends on the learning curve of the respective surgeon. This may explain the lack of reported difference between SLC and MLC patients in the present study because the participating surgeons were highly experienced and performed more than 50 SLC procedures each. This hypothesis is supported by the study results of laparoscopic cholecystectomy. After its introduction, Zucker et al. described a medium surgery duration of 118 min, which was reduced steadily thereafter [28, 29]. Therefore, the length of SLC surgeries approximates the duration of MLC surgeries with increasing experience.

The conversion rate within both groups was similar, which supports the results in the relevant literature [10]. In contrast, Haueter and colleagues and Evers et al. found a significantly higher necessity of additional trocars in the SLC group, but the conversion rate to open cholecystectomy was similar in both groups [9, 26]. Omar et al. defined success as surgeries that were completed without additional trocars or conversion to open cholecystectomy. He referred to significantly higher success rates in the MLC group and a higher necessity for conversion in the SLC group. If the first ten cases are set aside, then the conversion rates in both groups were adjusted [19].

The prolonged duration of surgery and the higher conversion rate suggests that some technical difficulties are associated with the SLC technique. Several studies examined the question of difficulty of the SLC technique and concluded that SLC produced more discomfort [18] and involved a more challenging handling of the instruments [24] and a higher level of exposition of the gallbladder [30], physical exertion and stress [31]. The preparation of the gallbladder was reported as equally difficult [24, 30], but the removal was easier in the SLC group compared with the MLC group [24]. Some disadvantages in the handling of the instruments due to the lack of triangulation were stated [17, 23, 25, 32, 33]. Surgeons in the present trial evaluated the difficulties of the surgical access, the preparation of the gallbladder hilus and the preparation of the gallbladder fossa. No differences in these difficulties were found between the surgical techniques. Notably, the occurrence of a learning curve is mentioned in the literature, but only experienced surgeons participated in the present study. Analogous to the duration of surgery, the conversion rates exhibit comparable feasibility with increasing experience of the surgeons [27].

The present study found no significant differences in the occurrence of intraoperative complications. Bile duct injuries are a rare but particularly feared complication in gallbladder surgery that highly impact postoperative morbidity [34, 35]. An increased incidence of bile duct injuries was not supported in the present study, but it was reported by some authors [36, 37]. Recent studies presented comparable results of a similar occurrence of bile duct injuries using both surgical techniques [9, 10, 17, 19, 21, 38]. Arezzo et al. examined the occurrence of complications in a recently published meta-analysis and observed a higher complication rate of severe complications (Cavien-Dindo ≥ 3) in the SLC group, which is similar to that in Evers et al. and Saad et al. [7, 24, 26]. However, there was no difference in bile duct injuries. However, a larger patient collective than that in the present study or meta-analysis is required to make a reliable statement of the real rate of rare complications, such as bile duct injuries.

SLC patients in the present study had a significantly shorter hospital stay compared to MLC patients. Recent studies only partially support these results and showed a lower [10, 25] or equivalent [7, 9, 17–20] length of hospital stay for patients in the SLC group. Even though the results from the present study with 3 to 4 days of hospital stay appear long at first sight, it represents our daily routine because elderly or critically ill patients as well as patients with acute inflammation or previous abdominal surgery were not excluded in this study and might therefore prolong the mean hospital stay. However, the length of hospital stay depends on a variety of factors, such as the occurrence of complications, pain intensity and hospital policies. Therefore, comparisons of the duration of hospital stays across geographical and cultural regions and health systems are highly questionable. For more than a decade, a discussion is ongoing whether laparoscopic cholecystectomies can be performed as day-surgery surgeries. An updated review of the Cochrane Database and a recent meta-analysis conclude that it appears to be as safe as overnight laparoscopic cholecystectomy in selected patients, but the overall effectiveness for the patient is still unclear [39, 40]. Besides the guidelines by the Society of American Gastrointestinal and Endoscopic Surgeons (SAGES), also, an Italian working group recently stated in their consensus conference guidelines that elective day-surgery laparoscopic cholecystectomy can be considered in carefully selected patients [41, 42].

Regarding the primary endpoint of the present study, we found a tendency of less pain on the morning of the first postoperative day in the SLC group, but without statistical significance. The VAS pain score on the day of the patients’ discharge from the hospital was significantly in favour of SLC. Postoperative pain in the literature is controversial. A significant limitation in the comparability of techniques is the use of different follow-up periods and considerable heterogeneity in results and methodologies between the individual studies. Overall, the measurement of postoperative pain in the relevant literature was not standardized, and it was performed at different times. Although the benefits of SLC are not supported consistently in the literature, a trend towards lower pain levels after SLC was observed. Arezzo et al. showed significantly lower pain intensity in the SLC group, but the results in the individual studies analysed were measured at eight different time points [7]. Haueter and colleges demonstrated significantly lower pain intensity in the first 12 h after SLC surgery, but statistical significance diminished at all other observed time points of pain follow-up. After 7 days, a trend of higher pain levels in the SLC group was reported [9]. Tamini et al. showed significant advantages in postoperative pain intensity in favour of the SLC technique after 24 h, which is supported by our results [10]. Further randomized studies confirmed the improved pain relief after SLC compared with MLC [8, 18, 20, 43], but others showed no difference [17, 19, 21]. Some studies suggested more postoperative pain [22, 23, 38]. The hypothesis was that pain would result in an enlarged fascial incision at the navel, which is the most painful incision in the process [44]. Removal of the first ten surgical observations from Deveci et al. revealed a significant difference [23]. These results suggest that the pain reduction was due to improved surgical experience, and a decrease in operative trauma resulting from a lower mechanical force and strain of the tissue [45]. Very few data are available on chronic pain following SLC in comparison with MLC. Christoffersen et al. did not find a difference in chronic pain [14], which is consistent with our results.

Another factor in patient’s postsurgical satisfaction is their general contentment with the cosmetic result of the surgery. The relevant literature illustrates consistency in this regard and shows the benefits of the SLC technique [7, 8, 10, 17, 18, 20, 22, 23, 26]. However, most studies included results only up to 12 months postoperatively. The present study found no difference between SLC and MLC in the cosmetic results approximately 6 years after surgery. These findings are consistent with the long-term cosmetic results. Bencsath et al. claimed that the cosmetic benefits were overrated and showed that patients forgot the number of trocars that were used after 21 months [44].

The cost-effectiveness of SLC is still a subject of ongoing debate. Two randomized trails report results regarding the cost of the operation. Bucher et al. found the cost of SLC to be higher in a Swiss trial, and Pan et al. from China report equivalent operative costs compared to MLC [8, 20]. In retrospective studies, the results differ from lower to substantially higher cost for SLC [46–49]. One of the main cost factors in SLC is the use of a disposable commercial port system which can be significantly reduced by the usage of a reusable port as reported by Shussmann et al. [49].

The widespread view is that the greater length of the naval incision in the SLC technique increases the risk of developing an incisional hernia [38]. Various studies showed that most incisional hernias after MLC occurred in naval incisions with the most extensive fascial cut [14, 50, 51]. Several recent meta-analyses showed a higher hernia rate in the SLC group [7, 9, 12]. In the three mentioned meta-analyses, the high hernia rate may be traced back to a single RCT with a significant impact and a particularly high level of incisional hernia rate [38]. If this single study is excluded, the differences in incisional hernia rates are similar, according to Antoniou et al. [12]. Haueter and colleagues performed a subgroup analysis without this particular study of Marks et al. and no longer found a significant difference in incisional hernias [9]. Arezzo et al. discussed the high rate of incisional hernias in the respective study with particular attention to the high occurrence of superficial wound infections as a risk factor for incisional hernia occurrence [7]. Antoniou et al. noted that there was a lack of experience in the surgeons performing SLC in this particular RCT [12]. When considering only studies with three-port MLC, there was no difference in hernia rate compared to SLC [7]. The comparatively low overall rates of incisional hernia should be emphasized as 2.2% and 1.3% in the SLC group and 0.7% and 0.3% in the MLC group. These hernia rates are lower than those in the present trial. The reason for this difference may be the difference in follow-up length. The studies included in Antoniou et al. observed their patients for up to 1 year. Haueter et al. showed a maximum follow-up of 69 weeks, and the meta-analysis of Arezzo et al. did not mention the follow-up length. A follow-up of 2–3 years is recommended to elucidate the real risk for incisional hernia [14]. Very few studies fulfilled these recommendations, including a study of the Danish National Patient Registry with a mean follow-up of 48 months. Christoffersen et al. demonstrated similar hernia rates of 4% in the SLC group and 6% in the MLC group [14]. Julliard et al. analysed the occurrence and risk factors for incisional hernias and showed a hernia rate of 7.9% after a mean follow-up of 41 months [52]. Bury et al. examined the contradiction in occurrence of incisional hernia and compared the works of Antoniou et al. and Christoffersen et al. He recognized both studies as equivalent in their level of evidence and stated the need for long-term investigations of this topic [13]. We can make an essential contribution with our almost 6-year follow-up period. The present trial detected similar hernia rates. The hernia rate was 5.7% in the SLC group and 8.3% in the MLC group. Therefore, the hernia rate described by Christoffersen et al. is supported by our results with an even longer follow-up period of 70.4 months on average. There were several risk factors mentioned in the relevant literature, especially the duration of surgery, the intraoperative manipulation and the method of fascia closure [53, 54]. In many cases, the importance of fascia closure in connection with the longer incision and hernia occurrence was stressed [9, 19, 32, 52]. Because only experienced surgeons participated in the present trial, a short duration of surgery, minimal intraoperative manipulation and a standardized facial closure are assumed. This result suggests that the incisional hernia rate does not differ between the surgical techniques using experienced surgeons and a standardized fascial closure.

There are some other possible operative alternatives to SLC regarding the minimization of the incision. Mini-laparoscopy using 3- to 5-mm trocars is a widespread alternative to standard MLC [24]. A meta-analysis comparing different kinds of laparoscopic cholecystectomy found four-port mini-laparoscopy favourable regarding the highest cosmetic score and the lowest postoperative morbidity, and SLC favourable regarding the lowest postoperative pain and shortest hospital stay [55]. Another alternative that can even be performed under local anaesthesia as day-care surgery is a small-incision cholecystectomy through a cylinder retractor. Grau-Talens et al. showed in a large prospective study that this operation is safe and feasible in three out of four patients with cholelithiasis [56].

The present study has some limitations. Neither the postoperative care surgeons nor the patients were blinded in our trial. Just recently, the Study Centre of the German Surgical Society formulated recommendations for the use of blinding in surgical trials that should be considered in future trials [57]. Another point of limitation is that we did not assess information on the exact extension of the umbilical incision to recover the gallbladder in the MLC group. In some cases, especially with large gallstones, it is necessary to widen the fascial incision to securely recover the gallbladder from the abdominal cavity. This need may even widen the fascial incision in MLC to the size of the incision made in SLC procedures. Another point of criticism is the high lost-to-follow-up rate in this study, which was probably due the long follow-up period. Furthermore, the patients were only subjected to a physical examination when they were interested in such an examination or reported discomfort. Therefore, a real hernia rate higher than that reported in the study may not have been found due to the lack of detection of asymptomatic hernias rather than their non-occurrence.

## Conclusion

The present study demonstrates that SLC is a safe and feasible alternative to cholecystectomy in experienced surgical hands with short-term advantages in cosmetic results, postoperative pain and length of hospital stay, but the advantages regarding cosmesis and pain do not persist in the long term. Almost 6 years after surgery, no difference in pain and cosmetic scores and incisional hernia rates between SLC and MLC were noted.
